# “*Doctors are targeted and kidnapped*”: crimes and insecurity contribute to health problems and constrain the delivery of health services in urban settings in Nigeria

**DOI:** 10.3389/fpubh.2025.1671252

**Published:** 2026-01-12

**Authors:** Tochukwu Charles Orjiakor, Ethelbert Agu, Prince Agwu, Pamela Adaobi Ogbozor, Divine Ndubuisi Obodoechi, Chidiogo Orjiakor, Aloysius Odii, Chizoba Ugwuoke, John Eze, Nicola Bowes, Obinna Onwujekwe

**Affiliations:** 1 Department of Psychology, University of Nigeria, Nsukka, Nigeria; 2 Health Policy Research Group, University of Nigeria, Enugu Campus, Enugu, ON, Nigeria; 3 Department of Psychology, University of Toronto Scarborough, Toronto, ON, Canada; 4 Department of Social Work, University of Nigeria, Nsukka, Nigeria; 5 Department of Social Work, University of Dundee School of Humanities Social Sciences and Law, Dundee, United Kingdom; 6 Department of Psychology, Enugu State University of Science and Technology, Enugu, Nigeria; 7 Department of Economics, University of Nigeria, Nsukka, Nigeria; 8 Department of Biochemistry, University of Nigeria, Nsukka, Nigeria; 9 Department of Sociology/Anthropology, University of Nigeria, Nsukka, Nigeria; 10 Cardiff School of Sport & Health Sciences, Cardiff Metropolitan University, Cardiff, United Kingdom

**Keywords:** crime, health, health facility, insecurity, urban

## Abstract

**Background:**

Nigeria ranked third in Africa and eleventh globally in the 2024 Crime Index. Despite the country’s endemic crime level, its impact on the health sector has been under-researched. The paper presents qualitative data from a study conducted in selected urban Local Government Areas in Abia and Anambra states in the southeastern part of Nigeria.

**Method:**

Fifty-two key stakeholders in security and health sectors, comprising health policymakers (*n* = 8), public safety officers (*n* = 10), local community leaders (*n* = 10), frontline health managers (*n* = 14), and informal healthcare providers (*n* = 10), Eight focus group discussions were held with male and female service users.

**Results:**

Findings indicate that acquisitive offenses such as theft, robbery, kidnapping, sexual offenses, and gang-related or cultism-driven violence were frequently reported. Residents, healthcare professionals, local authorities, and policymakers noted that these crimes posed significant threats to health workers and negatively impacted the functionality of health facilities. Incidents of staff absenteeism, equipment theft, and night shift interruptions were reported. Furthermore, elevated crime rates have led service users to consider safe times when accessing healthcare facilities carefully.

## Introduction

In rapidly urbanizing low- and middle-income countries (LMICs), rising crime rates are often linked to imbalances between population growth, resource availability, and essential services (1). Over half of Nigeria’s population now lives in cities, placing it third in Africa and eleventh worldwide for crime levels (2, 3). High crime rates impact physical and food security as well as the economy, drawing national and regional attention (4, 5). Although crime, fear of crime, and related health issues are connected, there is limited qualitative evidence on how crime affects illness burden and access to health services (6, 7), especially in low-resource contexts. Examining the impact of crime on health in urban environments may contribute to progress on Sustainable Development Goals (SDG) 8 (decent work and economic growth), 11 (sustainable cities and communities), and 16 (peace, justice, and strong institutions).

Nigeria is facing various forms of organized crime, including terrorism, banditry, militancy, cultism, kidnapping, human and drug trafficking, and armed robbery (8, 9). The primary crime and security issues differ between northern and southern regions. In northern Nigeria, terrorism and banditry are prevalent (10), often targeting rural isolated communities, while political militancy and cultism are more commonly reported in southern Nigeria (11). Kidnapping and armed robbery are crimes that occur across the entire country (12). Urban areas experience various spontaneous, crimes, including trafficking, substance misuse, theft, unlawful possession of arms, and rape (13, 14). Typically, these criminal offenses are more prevalent in cities than rural areas, highlighting the importance of discussing their impact on health (15, 16). Despite rising crime rates and insecurity in Nigeria, there is limited discourse on how these concerns affect public health.

There is substantial evidence linking high crime levels to poorer health outcomes (17, 18). Recent health reports in Nigeria have called for a national security policy and services for health facilities, highlighting increasing crime-related concerns (19, 20). While we herald these reports, we acknowledge that they have realized that they fell short of recognizing the robustness of the issue as extending to the health of populations, and not just access to health facilities. To commence a robust enquiry into this issue, we focus on urban areas in Nigeria, notably because of evidence supporting an exponential rise in cases of urban crimes in the country (16, 21). We intend to present the nature of crimes and their impact on health in urban areas of Nigeria. We also hope that the continuously emerging field of urban health will benefit from our approach to highlight the often-neglected topic of crime and health challenges. This has contributed to the living challenges facing urban areas, with knock-on effects (22, 23).

Health authorities in Nigeria have focused more on navigating spaces for health services delivery in typically remote/isolated areas in the northern region, most affected by terrorism and banditry, but have offered almost no attention to the increasing rates of crime and insecurity happening in urban communities (24). Narratives on local strategic health documents and the newly inaugurated Sector-Wide approach (SWAp) do not seem to acknowledge the realities of increasing crime rates and associated impacts on health [e.g., (25, 26)], despite acknowledging the need for intersectoral partnerships. This affirms the need to put forward more convincing evidence on the effects of crime on population health.

Nevertheless, there is evidence that governance actors are responding to security problems by setting up security structures at least in public health facilities, thus acknowledging the reality and significance of security/crime challenges (19, 27, 28). However, literature exploring the nature and characteristics of the security/crime threats faced by urban residents and health facilities in the country and subregion is scarce. Rising concerns of crimes in urban settings, especially in LMICs, means that urban health studies must be expanded to capture such. Therefore, in addition to discussing physical safety for the delivery of health services, the current study presents new insights into how crime increases burden of illnesses. To underscore these issues, we will be addressing the following questions, (a) What are the common types of crime threatening health and health service delivery in urban settings in Nigeria? (b) How do such crimes affect health concerns? And (c) What responses are obtainable locally to sustain health services in urban settings affected by crime?

## Materials and methods

The current study is part of a larger study that aims to deeply explore the nature of crimes in and their impact on health in urban places, especially in low-resource contexts. Here we adopt a qualitative approach in presenting our inquiry into crime and health in urban spaces. We adopt both a positivist and constructivist research paradigm to adequately present the objective reality as narrated by relevant stakeholders. We then take a step further to construct or interpret objective experience. We adopted the Standards for Reporting Qualitative Research (SRQR) (29) guidelines to present our study.

### Researchers’ characteristics

Our research team (5 males and 4 females) is made up of a multidisciplinary team of health system researchers, including psychologists, sociologists, social workers, physicians, and economists. Five of the researchers had doctoral degrees, 3 had master’s and 2 were postgraduate students at the time of the study. Aside from the researchers who were postgraduate students, other research team members had a minimum of 5 years experience in qualitative research and had been team members in previous research projects. The lead researcher had postgraduate training in forensic and clinical psychology and so is knowledgeable in the areas of crimes and offending behaviors.

#### Study area

Our study was conducted in two major urban cities in the southeastern part of Nigeria. We selected two strategically important cities in the south-eastern region- Aba in Abia State, and Onitsha in Anambra State. Both cities are top economic hubs in the geopolitical region and have vibrant economic activities that tend to attract diverse crimes. These cities also have historical social events that allude to the prevalence of criminal activities that warranted the emergence of controversial local vigilante systems (30, 31). Recent reports still suggest considerable crime rates in both cities (32, 33). Nigeria’s health system is designed uniformly across the subnational with leadership mainly coming from the Ministry of Health and State Primary Health Care Development Agency. Communities rely more on primary healthcare facilities which are closest to them. They also get healthcare from private clinics that mostly provide primary health services. So, the health system structures in Aba and Onitsha are quite similar.

#### Study design and participants

We adopted a phenomenological qualitative research paradigm in the current study, seeking to understand the lived and perceived experiences of concerned stakeholders, including (but not limited to) urban dwellers/ health service users, service providers, around the relationship between crime and health (34). Our choice of phenomenological design was influenced by the need for exploration of the subject, which is relatively underexplored in the urban health literature. Thus, we designed our questions to elicit deep reflections touching on personal or third-party experiences.

Participants were purposively sampled from Abia and Anambra states in the Southeast region of Nigeria: Aba in Abia State and Onitsha in Anambra State. To reflect experiences across diverse stakeholder levels, select participants included state level policymakers from the health (*n* = 8) and public safety sectors (*n* = 10); identified neighbourhoods chairpersons and leaders of security outfits within neighbourhoods (*n* = 10), and formal (*n* = 14) and informal (*n* = 10) health providers within the covered neighbourhoods. Half of these participants came from either cities/states. Eight focus group discussions (*N* = 38, 4 male and 4 female groups) were conducted with community members across the cities to represent the experiences of urban residents and service users. Participants were aged between 28 and 75 years and all participants have had at least a primary level education.

### Ethical considerations

The study was approved by the Ethics Committee of the University of Nigeria, Teaching Hospital, Enugu State, Nigeria (NHREC/05/01/2008B-FWA00002458-1RB00002323) and the University of Leeds, United Kingdom (MREC 23–014). The study was also in accordance with ethical guidelines as outlined in the Declaration of Helsinki. Participants who consented to participate in the study signed consent forms. Respondents freely consented to participate in the study and were assured of anonymity, confidentiality and freedom to withdraw their consent at any point during the study or afterwards. Permission to audio-record the interview was also obtained from the respondents.

#### Instruments and data collection

In-depth interview and focus group discussion guides were utilized in the data collection process. The guides were pretested in neighboring states in the same region (Enugu and Imo) to assess respondents’ fatigue, coherence, and consistency with phenomenological insights in eliciting nuanced lived experiences. The instruments were designed using insights from the literature review and the experiences of some of the researchers who are locals in the study areas. They were administered by experienced qualitative researchers with terminal degrees, supported by graduate-level research assistants. The interviews were designed to last 1 h, while each group discussion was managed within 1.5 to 2 h. Informed consent forms detailing the full disclosure of the study and statements about confidentiality and anonymity were shared and signed by the participants before the commencement of data collection.

Participants were allowed to express themselves in English and/or Igbo, and the researchers were proficient in both languages. We ensured that interviews and group discussions were conducted in isolated locations where participants felt safe, secured and relaxed. Participants voluntarily participated in the study and were urged to refrain from mentioning names or identities if they felt unsafe or uneasy. They responded to our three main enquiries of (a) common crime scenarios constraining health services delivery in urban settings (b) connections between these crimes and increased burden of illnesses, and (c) locally obtainable responses that sustain health services in urban settings affected by crime.

#### Data analysis

Thematic analysis (35) was used to analyze the transcribed texts. This enabled the researchers to identify, code and classify themes in the transcripts, leading to a clearer grasp of the respondents’ experiences with respect to crime and health. The key themes were identified and extracted across the 60 transcripts, as well as salient quotes that serve as evidence for the themes (Table 1).

**Table 1:** Summary of crimes, impact on health services, and responses in urban Nigeria.

Type of crime	Impact on health services and community	Responses / interventions
Physical assault and armed robbery	Health workers and residents are targeted; leads to early facility closure, avoidance of certain facilities, and fear.	Neighborhood vigilante groups; early closure of facilities; increased caution in accessing facilities.
Theft, burglary, acquisitive offenses	Disruption of services, loss of equipment, staff absenteeism, reduced 24-h service, increased fear among workers.	Installation of security measures; collaboration with vigilante groups; avoidance of night shifts.
Kidnapping/abduction for ransom	Facility closures, increased security measures, psychological trauma, posttraumatic stress, and sometimes fatalities.	Screening of facility visitors; closure of gates; community vigilance; reporting suspicious activity.
Cultism and gang-related violence	Health workers avoid certain areas, disruption of immunization and outreach, deserted neighborhoods, service disruption.	Avoidance of high-risk areas; scheduling outreach for safer times; community engagement for safety.
Drug-related offenses	Crime hotspots near health facilities, increased violence, challenges in policing, and community safety concerns.	Community vigilance; reporting drug hotspots; collaboration with local security outfits.
Sex abuse/crimes	Victims require medical care, health workers face challenges in reporting/follow-up, increased burden on health system.	Provision of medical care; confidentiality for victims; selective reporting to authorities.
Violent mortality	Avoidance of affected routes/facilities, disruption of access to care, fear of both violence and police raids/arrests.	Avoidance of affected areas; waiting for calm before resuming services; community alerts.
Domestic abuse and political violence	Bodily harm, injuries, deaths, disruption of daily life and health services, food insecurity due to disrupted farming.	Community support; local awareness; adaptation of farming and service schedules; vigilance during unrest.

## Results

### Types of crimes experienced to be constraining health services

Various types of crime were identified in both study areas. The most common were physical assault, armed robbery (commonly stealing and snatching of phones and handbags), drug-related crimes, burglary, kidnapping for ransom, rape, domestic abuse, transport-facilitated crimes, homicides, and gang-related crimes (a.k.a cultism). These criminal and violent activities were often carried out with weapons, and sometimes by intimidation.

#### Theft, robbery and other acquisitive offenses

Acquisitive crimes dominated participants’ experiences. Most crime experiences were in the form of perpetrators forcibly demanding valuable items from victims, which included both residents and health workers. Oftentimes victims are intimidated, threatened and coerced to give up valuables. Weapons (e.g., guns and knives) are often reported to be involved in such crimes.

*“He then raised his cloth and showed me a gun, then I threw the bag to him and he searched out every content in the bag, including collecting my phone. For some time, I avoided the facility […]”* (IDI, Female OIC, Aba).

Theft reported by respondents included petty theft of personal belongings, car theft, burglary into shops and business spaces to loot goods, and also breaking into health facilities to steal medical equipment and resources. Health workers reported being targets of crimes and violence. Although theft/robbery incidents were reported to occur both at night and daytime, the former were more common. The outcome is typically that health workers close early in the evening to avoid being targets. Service users also avoid facilities where theft is reportedly common mainly when they are located on in lonely areas.

Health facilities experienced being burgled and vital equipment were carted away by criminal elements. The fear of invasion of health facilities by criminal elements disrupted night shifts and made the health workers who were largely women to fear being raped.

*"Our laboratory equipment were stolen. These attacks on our facilities have affected our work. Health workers refuse to sleep in the facilities. Normally they should give twenty-four hours services, but nobody is ready to stay in a place where you are not sure that your life is safe. So, it has affected them giving twenty-four hours services"* (IDI, Female Health Secretary, Aba).

Theft and burglary targeting local facilities and stealing equipment point to the disruption of services that could be otherwise offered and further validate the fears of health workers that health facilities are unsafe places, especially for night shifts.

#### Kidnappings/abductions for ransom

Reports of kidnappings were common and could come in different ways. Although perpetrators targeted affluent people in the local community, random attacks were also reported, and anyone can be a target, irrespective of social status. One unique and commonly reported manner of crime operation is ‘one chance’ operators. Unsuspecting commuters board buses/taxis operated by crime syndicates, who then proceed to rob and harm victims when vehicles get to isolated areas where they will not be suspected:

“*those people you know commuters, picking people on what we call one chance. You will think you have entered a safe vehicle they take only you and maybe a few others before you know it they head for the bush*” [IDI, Director of public health, Anambra state].

Health workers, particularly doctors, were systematically targeted as they were considered affluent. One health worker described kidnappers coming in the guise of patients and caregivers and kidnapping a doctor on duty. This led to the facility closing its gates and sizing up potential ‘service users’ to detect any unusual demography (e.g., a group of young men coming in together) before opening up the facility. Different kinds of health loss arise from kidnappings. Perpetrators often demand large sums for ransom, and victims reportedly suffer severe beatings and threats to their lives, leaving many victims and their family members with posttraumatic stress symptoms. Cases of victims being killed either for esoteric ritual purposes or their organs being harvested were also reported. The crime is so common and dreaded that urban residents adopt any approach to avoid being victims.

#### Cultism-gang-related violence

Cult groups are ubiquitous violent gangs that evolved from youth activities within Nigerian tertiary education institutions, and have, more recently, expanded to the streets and communities across the country. The narratives of respondents suggest that cult activities have spread to much younger people, even in secondary-level education. Young people on the streets have increasingly adopted cult-related violence, typically styled by clashes between cult/gang groups. Cult clashes are typically characterized by killings and/or aggravated assault resulting in grievous bodily mutilation of victims.

*“The community is filled with cult groups …the health workers avoid some of the streets during immunization or they go to those places with lots of cult groups without their devices […]”* (FGD, P7 Male, Aba).

When gang/cult groups clash, it is almost certain that serious injuries and, in many cases, deaths would occur. The neighborhoods affected, as described earlier, are deserted, and when they are near a health facility, workers and service users are disrupted.

#### Drug-related offenses (use & trafficking)

The *dealing* and use of illicit substances was also commonly identified as a common crime-related experience in the studied urban spaces. Residents report experiencing an increased rate of substance use and consider the use of substances a crime, and acknowledge substance use to be a key driver of other criminal activities. It is important to mention that methamphetamine and cannabis were the commonly described drug types that were associated with crime in both cities.

*"Smoking [cannabis],…mkpuru mmiri [methamphetamine]after they might finish smoking the next thing they will think how to go and rob* [IDI, male, President general Onitsha community].

With insufficient and inadequate policing of the streets, deviant individuals and groups often spot or locate areas within neighborhoods where they access drugs of abuse or have networks that supply illicit substances of abuse. It is not unusual for crime and violent activities to happen in such places. Illicit drugs were particularly viewed as a direct facilitator of criminal and violent activities within urban neighborhoods in the current study.

#### Sex abuse/crimes

Sex crimes were common. Children who remain at home when parents/guardians go to work, domestic sex violence, and kidnap/armed robbery victims were the commonly described instances where sex crimes and abuse happened.

*Yes, the girl is living with someone and she was raped. She was brought to the health post, we did HIV test, cleaned the vulva, then gave her antibiotics, we checked if there is any where to suture or any bruises*. [IDI, female OIC, Onitsha]

Many of the victims of these crimes turn up for treatment in health facilities, and health workers shared the difficulty in engaging and navigating such victims. The uncertainty about which case to report, whether the victim wants to report, and how to follow up on the violations with the authorities can be fleeting. Health workers often end up attending to the immediate health needs of such victims, not beyond that.

#### Violent mortality

When violence erupts in communities, violent deaths are reported by the respondents to directly impact health and access to health services. Notably, a respondent narrated how a young boy was stabbed and killed and people are afraid of accessing the road where it happened.

*“…so they used keke to block the road and immediately they had weapons on them, chasing everyone …they stabbed a guy who was bleeding in excess. Everybody was running…the boy later died. A lot of people avoided that route for a long time, and it is the route that leads to the health facility”* (FGD, P2 Female, Onitsha North).

When homicides happen, it is not unusual for activities around the area are typically disrupted and the surroundings deserted before the law enforcement officers arrive- if they arrive. Residents remain indoors, businesses shut their doors, and people avoid these routes. The fear is not only because of the risk of more criminal activity, but also fear that law enforcement personnel are popular for raiding such areas and making random arrests. When this happens near a health service-providing facility, both service users and health workers avoid the area for a considerable amount of time until they are certain calm has returned and no police raids are underway.

*“I was in labour, and I was looking for means to go to the hospital but because of I think there was a robbery attack in that my area, and police was doing mass arrest, I could not get any vehicle to take me to the hospital […]”* (FGD, P5, Female, Aba).

The fear of being harmed by ongoing violence is almost equivalent to being arbitrarily arrested by police, as there is a crisis of confidence and irking distrust in the police organization. People feared being coerced to confess to crimes, being extorted, or even worse, when they are in police detention.

#### Other violent crimes- domestic abuse and political resistance-related violence

Family violence, especially intimate partner violence, but also violence against children, was commonly reported in our data set across the cities. Typically, women and children were the victims, and serious bodily harm or injuries were commonly reported.

…*where a the family have misunderstanding …they often beat the mother in the home, or also beat the child, you know stubborn children where the father is trying to correct the child they break the hands* [IDI, male Bone-setter, Onitsha]

Corporal punishment is common in the country, and it is not hard to notice situations where beatings go overboard, causing severe bodily harm, mutilations, or even deaths. Very commonly, intimate partners suffer from these domestic situations as well as children within households.

Also, because the study area was experiencing political strife as a result of agitations by government-proscribed separatist groups, spurts of violent activities disrupted daily living and affected health-related activities.

*this kind of insecurity we’re having in the eastern region or in Nigeria, this issue of ehm IPOB closure, sit at home, one thing or the other, seriously that one is affecting uhh us or every other health services.* [IDI, PMV coordinator, Onitsha].

At the time of the current study, cessation strife has resulted in the grounding of daily lives on Mondays- typically known as ‘sit-at-home’ as the leader of a faction of the separatists was being held and charged for various offenses by the federal government. On Mondays, business and commercial activities, including schools, mostly remain shut down in many cities in the southeastern part of Nigeria. People who defy the sit-at-home by coming out for travel or going to their various occupations risk being mugged or lynched by sympathizers of the agitators or some other criminal groups who thrive in the chaos. Other crimes that were reported include cyber fraud, and speeding/dangerous driving.

One interesting health impact is on local city farmers who predominantly engage in small-scale farming in the suburbs and depend on the city’s labor supply. On one hand, farming activities are disrupted by the sit-at-home days, as perishable agricultural produce spoils because farmers’ supply chains are disrupted. On the other hand, farmers experienced labor shortage because those who should help in the farms were rendered unproductive due to increasing drug addiction problems, mostly affecting youths. The consequence of these disruptions is a short food supply, which worsens an already existing food security crisis in the country.

*"These boys that get involved in this hard drug, they don’t have business coming to till the land for you, coming to clear the bush for you, no they don’t do these things. You will now see that the farmer who is aged and cannot cultivate two hectares of land alone. He may have the money to pay these labourers, but there are no labourers to pay. So, we cannot produce enough food. Even visiting the farms becomes a problem when the community becomes too hot with fights, killings, and clashes"* (IDI, Male WDC 1, Aba).

### Measures taken to address crime

#### Neighborhood watch/vigilante groups

Interestingly, the local police stations and personnel enjoyed little trust in the communities. Vigilante/neighborhood watch groups were perceived as closer and more responsive to the community’s security needs. Neighborhoods organized local vigilante groups to combat crime. Each urban neighborhood organizes and establishes a team of mostly young males to handle security matters within.

*“But these are the reason why my community engage …. community vigilante…because of the vigilante we formed through the collaboration with the police”* (IDI, Male WDC 1, Aba).

Neighborhood watch groups are typically established in collaboration with local police units/stations. These groups would receive specialized training in security approaches and light weapons and typically operate to safeguard designated residential and business areas. When suspects are arrested by neighborhood watches, they are expected to hand them in to the police for further investigation and prosecution.

#### Military checkpoints

Persistent insecurity challenges led to military checkpoints in several areas around the cities. We also found that military installations around cities also had operations, such as ‘Operation match them with strength’, ‘Operation stop and search’, and ‘Operation understand who is who in the community’, which were some of the various operation strategies adopted by the Navy. The theme of these operations concur with the high crime rates perceived by residents. Residents also had phone lines to reach military personnel when incidents occurred.

*“We give our number that you will call us when crime happen or when crimes about execute. Then we promise them that every information will be confidential. One, we are not going to open up your name and the number used to contact us. So these are the proactive measure we are using to tackle insecurity…people have confidence in us”* (IDI, Male Navy Officer, Aba).

It was unclear whether these were official or personal phone lines. Residents were also assured of optimal confidentiality should they reveal information that could lead to the identification of crime perpetrators. People also do their best to retire from their places of work and occupations in time and avoid very early or nighttime.

## Discussion

The present study examined crimes and security challenges affecting health in the urban areas of Aba and Onitsha, two major commercial centers in Nigeria with a history of violent restiveness and security concerns. Various offenses such as theft, kidnapping for ransom, violent incidents, cultism/gang-related activities, sex-related offenses, and drug-related crimes were identified as contributing to health risks and limiting access to health services. Previous studies [e.g., (36, 37)] have also highlighted the overrepresentation of crime and unrest in urban areas. Crucially, our findings extend beyond mere crime prevalence and reveal their profound and multifaceted impact on residents’ health, health workers’ safety and functionality, and the operational integrity of health facilities in an African context. Additionally, the results showed that crime can negatively affect both the quality of and access to health services, consistent with findings from previous studies [e.g., (38, 39)]. Potential physical health outcomes include serious injuries, sexually transmitted diseases, cardiovascular conditions like high blood pressure and stroke, as well as increased rates of violent mortality and related traumatic stress. These findings are consistent with earlier research indicating a relationship between crime and reduced mental and physical health (40, 41). These results add to current knowledge in the interdisciplinary areas of public health criminology, and health security.

Acquisitive crimes such as theft, armed robbery, kidnapping for ransom, and burglary were prevalent, affecting both residents and health facilities in both study areas. Individuals lost money and belongings, while medical centers lost essential equipment, leading to increased fear, service disruption, reduced capacity, and poorer patient care. Health workers reported reduced confidence during night shifts due to concerns about equipment theft, contributing to increased workplace stress. The prevalence of acquisitive crimes reflects the ongoing economic challenges in Nigeria, highlighting the necessity for a multisectoral strategy to address crime and its associated health effects. The creation of economic and employment opportunities has been shown to reduce violent crime (42). Reports indicate that Nigeria’s official unemployment rate ranges from 4 to 33%, a disparity attributed to differences in statistical estimation methods. Furthermore, with more than 112 million individuals living in extreme poverty and increasing economic inequality (43), it is reasonable to infer that poverty plays a significant role in the prevalence of acquisitive crimes.

A thorough analysis of the characteristics of kidnapping, as identified in the present study, is essential. Kidnapping for ransom, along with less evident motives such as organ trafficking and ritual killings, has emerged as a significant concern and appears to have reached critical levels. These findings are supported by recent scholarly research (44, 45) and media accounts (46), which document the ongoing kidnapping crisis in Nigeria. Criminal syndicates, especially in the southern region of Nigeria, often target affluent or prominent individuals, including health professionals. Crime syndicates also exploit weaknesses in local transportation for opportunistic abductions—an approach popularly called locally “one-chance operators”—resulting in theft or even fatalities. The long-term psychological consequences for individuals who have experienced kidnapping, such as anxiety and posttraumatic stress disorders, are well documented (47). Additionally, community members may develop heightened fear and vigilance, which can occasionally lead to disproportionate reactions to otherwise neutral situations. These factors underscore the ongoing need for multisectoral collaboration in addressing the challenges posed by kidnapping. Emphasizing the relationship among crime, insecurity, and public health may foster the development of intersectoral policies to reduce criminal activity and safeguard health. Strategies to reduce opportunistic kidnappings in urban areas and health facilities are important. The transportation sector shows regulatory gaps. City transportation systems are often chaotic, and it seems that governance actors are mostly focused on generating revenue from operators. Poor oversight of taxi and bus operators allows syndicates to target victims–victims wound up in health facilities, often in emergency conditions, stretching and already weak health system. Research suggests that a more regulated transit system offers benefits, including lower crime rates (61). While solutions may be complex, identifying health risks linked to transportation can promote more multisectoral consultations, interactions, and collaborations aimed at public health protection.

Other crimes that were not directly acquisitive in nature were cultism, sex-related crimes such as rape, and drug-related crimes. ‘Cultism’ is a gang-like phenomenon, seemingly unique to Nigeria, involving confraternity groups that have endured and evolved for nearly seven decades. Originally intended as a vibrant opposition to colonial rule and instituted in universities to attract young educated members to the colonial struggles, Nigerian cult groups have evolved to become more clandestine and violent and have expanded onto city streets. Clashes between cult subgroups are becoming more common in Nigerian streets, impacting health and urban decorum, especially in the southern region of Nigeria (48). Cult clashes are often extremely violent, including mutilation and violent killing of victims (who are often but not exclusively opposing cult groups). These cult confraternities are considered deviant antisocial criminal groups and are outlawed. Respondents in the current study shared that cultism is currently a driver of morbidity and mortality in both cities studied and beyond. Recent studies have pointed to disruptions of cultism on food security and economic wellbeing [e.g., see (49, 50)] in Nigeria. Health facilities and health workers are also threatened and impacted by cult clashes. Despite efforts to discourage cult membership [see (51)], cult activities are resurging in urban areas and have contributed to many violent deaths and health service disruption noted in our study. Youth and community violence-reduction programs could help reduce cult or gang affiliations. Berdychevsky et al. (52) found that recreation programs that are attractive, well-structured and supervised, and include elements of coaching/mentoring were effective and prompted meaningful reappraisal among Chicago youth. Such programs could be adapted in the African context.

Other criminal activities, including sex crimes and drug-related crimes, were also reported. Sex crimes were typically committed by predators who target children at home when their guardians are off to their occupations. Drugs of abuse, especially cannabis and methamphetamine, were commonly identified to be used by crime groups and drive the criminal activities experienced within communities. Sex crimes are widely documented in Nigerian communities and are still poorly addressed by the local police and social services (53). Other studies have also identified that drug use problems are common across African communities and crime networks are typically involved (13).

We do not assert that the spectrum and nature of crimes affecting health are unique or particularly remarkable for the region studied. Indeed, there is evidence that violent crime rates is somewhat rising in several African regions (54) Most societies experience different types of offending or criminal behavior. In the context under consideration, we try to bring to the fore the health impacts as health facilities have been identified as frequent targets. Insecurity has been a recurring issue in recent studies on Nigeria’s health systems [e.g., (11, 62)]. However, current policy documents and initiatives often do not explicitly address the direct health implications of crime and insecurity. The Nigerian National Health Policy (55) and the Basic Health Care Provision Fund (BHCPF) (56) incorporate provisions designed to enhance infrastructure (for security) and workforce capacity, including support personnel. If effectively implemented, these policy frameworks have the potential to strengthen security within health facilities. However, recent evaluations suggest that minimum standards and funding commitments for the BHCPF have been met only to a limited extent, as facilities across the country were reported to achieve a median compliance score of 23% regarding adequate human resources (57). It is more important now that a ‘health in all’ approach be used to identify and engage the problem of crime and insecurity.

We believe that the need to address security and crime concerns in urban areas through multisectoral cooperation, acknowledging that these issues have direct implications for public health, is the key lesson of our study. Actors within the health, criminal justice systems, community stakeholders, and city governance actors are particularly in the eye of the storm from our perspective. Security operatives, especially the police, are a critical group to engage in solving crime and insecurity problems. However, involving the police should be done cautiously. First, the Nigerian police enjoys little trust and confidence by the public (58), and the evidence resonates in the narratives from the current study. Indiscriminate arrests, extortion, and other corrupt practices that characterize the police indicate that the Nigerian police are due for a genuine reform. People fear cooperating with them and do not trust them to serve justice when perpetrators are apprehended. Local neighborhood watch groups are more trusted and closer to the affairs of community members, and so may be a more practical key with regard to mitigating crime and insecurity across urban neighborhoods. Other studies have also found neighborhood watch groups to be responsive and active in urban spaces (59). However, a vibrant policing system is critical to integrate and coordinate proper security and feedback to society. There is evidence that grassroots approaches that involve community actors and place less emphasis on police engagement hold more potential in controlling drug-related crimes (60). More conversations and engagements, especially at the grassroots level, are needed to identify feasible interventions and policies compatible with low-resource contexts, as crime/insecurity driven by challenging economic situations continues to trouble the region.

### Limitations and suggestions

Heightened security concerns, arising from separatist agitation in the region at the time of the study, meant that several respondents were apprehensive of researchers and may be unwilling to share the depth of their experiences. Although with the explanation that the focus of the engagements was exclusively a research exercise, a stronger rapport was elicited and most fears defused, we must highlight the tension in the social space at the time of this study. It is possible that despite agreeing to participate, some respondents may hold back from sharing some of their experiences for fear of reprisal attacks from actors from the separatist groups in the various communities. However, with assurances of confidentiality and the reassurance that they could decide what to share, most participants flowed freely through the data collection process. We do not suppose that the experiences narrated by the respondents represent what is happening across the country or within sub-Saharan Africa. In fact, at the time of revising this manuscript, a new wave of kidnapping, targeting school children and rural communities, has taken dominance, particularly in the northwest and north central geopolitical regions of the country. There may be other realities that differ from the experiences reported here in other states, regions, or subregions. Household surveys and other methodological approaches may help deepen understanding of the magnitude and spread of crime and insecurity across the wider region.

## Conclusion

The intersection of crime and public health in urban Nigeria, as illuminated by this study, reveals complex challenges that demand urgent and coordinated action. Kidnapping, cultism, sex-related offenses, and drug-related crimes not only undermine the sense of security but also directly impair the physical and psychological well-being of victims, families, and communities. Health facilities and workers are increasingly vulnerable to these threats, causing disruptions to essential service delivery and contributing to rising morbidity and mortality. Our findings underscore the necessity of adopting a multisectoral response. Strengthening security protocols within health facilities, reforming the transportation sector to reduce opportunistic crimes, and implementing robust youth and community violence-prevention programs are imperative. Moreover, building trust through community-based approaches, such as engaging neighborhood watch groups while cautiously reforming policing strategies, is critical to restoring public confidence and ensuring justice. Addressing the deeply interwoven nature of crime and health challenges in urban Nigeria and all other similar low-resource contexts requires holistic strategies, persistent political will, and genuine community engagement. Additionally, crime prevention must be mainstreamed into health policy, recognizing that safeguarding health extends beyond clinical care to include the broader social and environmental determinants of well-being.

## Data Availability

The raw data supporting the conclusions of this article will be made available by the authors, without undue reservation.
