# Supplementary material for: “Doctors are targeted and kidnapped”: crimes and insecurity contribute to health problems and constrain the delivery of health services in urban settings in Nigeria
Source: Front Public Health. 2026 Jan 12;13:1671252. doi: 10.3389/fpubh.2025.1671252 (PMC12832293; doi:10.3389/fpubh.2025.1671252)
Supplement: Supplementary file 1 [file Data_Sheet_1.PDF]

Supplementary

In-depth Interview guide for health policy makers on the impact of crime on the health of urban residents

Introduction: Briefly explain the purpose of the interview and give the Information and consent form.

Ask for permission to record the interview. Record start and end date.

Demographic information of Interviewee:

Name of organization:
Cadre/Role:
Designation:
Gender:
Age:
Highest education level:

**Questions**	**Probes**
**Crime exposure/experience**	
1. Can you provide a brief overview of your job role and responsibilities as it pertains to health services in the state/LGA?	
2. What types of crimes have you experienced or heard about in the state/LGA?	Probe:<br>Violent, Domestic violence, Sexual, burglary, property etc<br>How often does these crimes occur?
3. How often do you encounter patients who are victims of crime or receive reports from your facilities on crime-related issues on crime in the state/LGA?	
**Impact of Crime**	
4. In what ways have these crimes affected you?	Probe for health, physical, psychological, emotional, financial and privacy
5. Have you noticed any patterns in the types of health issues among residents that arise due to crime in the neighborhood?	
6. How have these crimes affected the social relationships in the community?	Ask about how crime affects their relationships with their patients, colleagues, supervisors, and family.

7. How does crime in the neighborhood affect your work as a policy maker in health sector?	Health, well-being, performance, and motivation of health workers?<br>Ask about how crime affects their career aspirations, professional development, and retention.<br>Ask about how crime affects their income, expenses, and livelihood.
8. In what ways have these crimes reported affects provision of health services?	Probe for impact on health workers, facility (work times), service users
9. Who are more affected by crime in the community?	Probe for community members, security personnel, community leaders, perpetrators, victims, or relations to the perpetrator
**Managing/Responding to crime impact on health**	
10. How do you cope with the incidence of crimes in the LGA/state?	Ask about how they cope with the emotional and psychological effects of crime exposure.
11. What resources are available to help address challenges of crimes in the state?	Criminal justice system actors (police, vigilante etc), policies?<br>Ask about how they report or respond to such crimes, and what support or protection they receive from their employer, colleagues, or authorities. Probe for *counselling, legal aid, and financial assistance.*
12. Are there policies or programs is available to prevent or reduce crime in the health sector or in your neighborhood?	Ask about what challenges or barriers they face or perceive in implementing or accessing such policies or programs.<br>Probe for *counselling, legal aid, and financial assistance.*
13. Are there policies that suggest how to best respond to health problems (e.g. gun injuries) that arise from suspected criminal activities	
14. Who can help address the situation of crime in the neighborhood?	
15. From your interaction with the crime victims, how do they seek services to address their grievances?	
16. Who are the most affected (Children, youths, old, gender).	

17. Besides health sector, are there other sectors/factors that could help mitigate the influence of crimes on health in urban residents?	
18. What recommendations can you offer for reducing the impact of urban crime on health?	

Conclusion

- Thank the interviewee for their time and participation. Ask if they have any questions or comments.
- Inform them about the next steps of the research process and how they can contact you if they need any clarification or feedback.

In-depth Interview guide for security personnel on the impact of crime on the health of urban residents

Introduction: Briefly explain the purpose of the interview and give the Information and consent form.

Ask for permission to record the interview.

Demographic information of Interviewee:

Position in work:

Designation:

Gender:

Age:

Highest education level:
Type of security outfit: [Police, NSCDC, Vigilante]

**Crime exposure/experience**	
1. What types of crimes or security issues are reported to you in this area?	Probe:<br>Violent, Domestic violence, Sexual, burglary, property etc<br>How often does these crimes occur?
**Crime Impact**	
2. What kind of health problems are associated with each kind of crime?	Probe for physical, psychological, emotional, financial and privacy
3. In what ways are security personnel affected?	Physical, psychological/emotional, financial, privacy ?
4. How response to crime has affected your health and well-being?	

5. Are there any patterns in the types of health issues that arise due to crime in the neighborhood?	
6. How does crimes affect the health services in the area?	Health, well-being, performance, and motivation of health workers?<br><br>Ask about how crime affects their income, expenses, and livelihood.
7. How have these crimes affected the social and economic relationships in the community?	Businesses, social events etc
8. In what ways have these crimes affect your work in your security facility?	Probe for impact on health workers, facility (work times), service users
9. Who is most affected by crime in the community?	Probe for community members, security personnel, community leaders, perpetrators, victims, or relations to the perpetrator.
**Managing/Responding to crime impact on health**	
10. How do you cope with the incidence of crimes in your community?	Ask about how they cope with the emotional and psychological effects of crime exposure.
11. How do you handle situations where there is a clear connection between crime and public health issues, such as drug abuse or mental health crises?	Ask about how crime affects their relationships with their patients, colleagues, supervisors, and family.
12. What resources/training are available to you to help address challenges of crimes?	Criminal justice system actors (police, vigilante etc), policies?<br><br>Ask about how they report or respond to such crimes, and what support or protection they receive from their employer, colleagues, or authorities.
13. Can you share any success stories or examples where proactive security measures have significantly contributed to crime reduction and improved public health outcomes in the community?	Ask about what challenges or barriers they face or perceive in implementing or accessing such policies or programs.

14. How do security agents engage with residents to build trust and encourage collaboration in addressing both crime and health issues?	Are there community outreach programs in place?
15. Who can help address the situation of crime in the neighborhood	
16. How do security agents stay informed about emerging trends in crime and health, and how does this knowledge influence their strategies and decision-making in the community?	
17. Are there challenges or obstacles that security agents commonly face when trying to address the intersection of crime and health in urban settings, and how do they navigate these challenges?	
18. Is there any other matter you may want to share with us regarding the topic we discussed?	

Conclusion: Thank the interviewee for their time and participation. Ask if they have any questions or comments.

Inform them about the next steps of the research process and how they can contact you if they need any clarification or feedback.

In-depth Interview guide for health managers on the impact of crime on the health of urban residents

Introduction: Briefly explain the purpose of the interview and give the Information and consent form.

Ask for permission to record the interview.

Demographic information of Interviewee:

Position in work:

Designation:

Gender:

Age:

Highest education level:
Type of health facility: [Public/ private]

Level: Primary health?s

Staff strength: Services the facility provide:

**Crime exposure/experience**	
1. What types of crimes have you experienced or heard about in this area?	Probe:<br>Violent, Domestic violence, Sexual, burglary, property etc<br>How often does these crimes occur?
2. How often do you encounter patients who are victims of crime in your facility?	
**Impact of Crime**	
3. In what ways have these crimes affected you as a healthcare worker?	Probe for physical, psychological, emotional, financial and privacy
4. In what ways are patients affected?	Physical, psychological/emotional, financial, privacy ?

5. Have you noticed any patterns in the types of health issues that arise due to crime in the neighborhood?	
6. How have these crimes affected the social relationships in the community?	Ask about how crime affects their relationships with their patients, colleagues, supervisors, and family.
7. How does crime in the neighborhood affect your work as a healthcare provider?	Health, well-being, performance, and motivation of health workers?<br><br>Ask about how crime affects their career aspirations, professional development, and retention.<br><br>Ask about how crime affects their income, expenses, and livelihood.
8. In what ways have these crimes reported affects your health facility?	Probe for impact on health workers, facility (work times), service users
9. Who are more affected by crime in the community?	Probe for community members, security personnel, community leaders, perpetrators, victims, or relations to the perpetrator
**Managing/Responding to crime impact on health**	
10. How do you cope with the incidence of crimes in your community?	Ask about how they cope with the emotional and psychological effects of crime exposure.
11. What resources are available to you to help address challenges of crimes?	Criminal justice system actors (police, vigilante etc), policies?<br><br>Ask about how they report or respond to such crimes, and what support or protection they receive from their employer, colleagues, or authorities. Probe for *counselling, legal aid, and financial assistance.*
12. What policies or programs is available to prevent or reduce crime in the health sector or in your neighborhood?	Ask about what challenges or barriers they face or perceive in implementing or accessing such policies or programs.<br><br>Probe for *counselling, legal aid, and financial assistance.*

13. Who can help address the situation of crime in the neighborhood?	
14. From your interaction with the crime victims, are there ways they circumvent their crime(s) to achieve their goal(s)?<br>15. Who are the most affected (Children, youths, old, gender).<br>16. Besides health sector, are there other sectors/factors that influence health crime?<br>17. What recommendations can you offer for reducing the impact of urban crime on health?	

Conclusion

- Thank the interviewee for their time and participation. Ask if they have any questions or comments.
- Inform them about the next steps of the research process and how they can contact you if they need any clarification or feedback.

IDI with community leaders on the impact of crime on the health of urban residents

Introduction

Introduction: Briefly explain the purpose of the interview and give the Information and consent form.

Explain that the interview will take approximately 30-45 minutes.

Ask for their consent to participate in the interview and for the interview to be audio recorded.

Demographic information of Interviewee:

Occupation:

Position in the community:

Gender:

Age:

Highest education level:
Years of experience as a community leader.

Name of community:

State:

A: Experience with Crime in the Community

1. What crimes have you witnessed or experienced within your community?
2. How often do you hear about residents who have been victims of crimes?
3. What are the causes of crime in your community?
4. How does crime in the community affect the lives and livelihood of residents?
5. How does crime affect the social and economic activities of the community?

B: Impact of Crime on Health

6. How does crime in the community affects the health of residents?
    a. Probe for physical and mental health concerns
7. In your community, what types of health issues do you think/see arise due to crime in the community?
8. How does crime impact the finance of people in the community?

9. Who are more affected by crime in the community? Probe for community members, security personnel, community leaders, perpetrators, victims, or relations to the perpetrator.

C: Community Responses to Crime

10. How does your community respond to crime and safety concerns? (Probe for any response they have made in the past and plans for the future in response to crime)?
11. What programs or resources are in place to address the health impacts of crime in the community?
12. What are the available victim support services in the community? Probe for *counselling, legal aid, and financial assistance.*
13. What recommendations do you have for improving community responses to crime?
14. What recommendations would you suggest for addressing the health impacts of crime in the community?

D: Collaboration between Community Leaders and Healthcare Providers

15. How do community leaders and healthcare providers work together in addressing the health impacts of crime?
16. How effective has the collaboration between community leaders and healthcare providers been?
    a. What has worked and what has not worked?
17. What recommendations can you offer for improving collaboration between community leaders and healthcare providers?

Conclusion

- Thank them for their time and participation in the interview.

Ask if they have any additional comments or recommendations related to the impact of crime on the health of their local residents.

Focus Group Discussion Guide for Service users on the impact of crime on health in urban neighborhoods

Introduction

- Introduce yourself and explain the purpose of the focus group discussion.
- Explain that the discussion will take approximately 60-90 minutes.
- Ask for their consent to participate in the discussion and for the discussion to be recorded.
- Each participant will read and sign the consent form and provide demographic information including how long they have lived in the neighborhood. (this will later be anonymized)
- Participants are to introduce themselves with alphabets or numbers as identification codes and must mention the codes before responding to a question.

Experiences of crime within the neighborhood

1. What crimes have you witnessed or experienced or heard of within your neighborhood?

Probe: for variant types of crimes: property, burglary, violent, sexual assault?

Impact of Crime on Health

2. What patterns of health issues have you noticed arise due to crime in the neighborhood?(Probe for hypertension, panic attacks, chronic stress, insomnia)
3. How have these crimes in the neighborhood affected your physical health?
4. How have these crimes affected your psychological/emotional/mental wellbeing?

(Probe for depression, anxiety, mood swings, post-traumatic stress disorder, anger, fearful, low participation in physical and social life activities in the community and social life, isolation)

5. How have crime issues within your community affected you socially and economically?
6. How does crime in your neighborhood affect your access to healthcare services in your neighborhood?

Community Responses to Crime

7. How has your community responded to crimes and safety concerns within your neighborhood? (who acted? What was done?) ? (Probe for any response they have made in the past and plans for the future in response to crime)?
8. Are there any programs or resources in place to address the health impacts of crime in the community? (Probe for rehabilitation centers, health talks/campaigns)
9. What recommendations would you make to improve your community's responses to crime?

Collaboration between Service Users and Healthcare Providers

10. How are health service providers affected by crimes within the community?
11. How are health service providers prepared to respond to the impact of crimes on the health of residents in the neighborhood?
12. What recommendations do you have for improving collaboration between service users and healthcare providers to reduce crime and its health effects in your neighborhood?
13. What are the community approaches to preventing and addressing crime in your area?
14. How effective are these approaches?
15. What are the available victim support services in the community? Probe for *counselling, legal aid, and financial assistance.*
16. What recommendations do you have for addressing the health impacts of crime in the neighborhood?

Conclusion

- Thank them for their time and participation in the focus group discussion.
- Ask if they have any additional comments or recommendations related to the impact of crimes on health in urban neighborhoods.
